# Research Progress on the Anti-Disproportionation of the ZrCo Alloy by Element Substitution

**DOI:** 10.3390/ma13183977

**Published:** 2020-09-08

**Authors:** Mingde Wu, Jingchuan Wang, Peilong Li, Cun Hu, Xiaofeng Tian, Jiangfeng Song

**Affiliations:** 1 The College of Nuclear Technology and Automation Engineering, Chengdu University of Technology, Chengdu 610065, China; wumingde901129@163.com; 2 Institute of Materials, China Academy of Engineering Physics, Jiangyou 621908, China; wangjingchuan@caep.cn (J.W.); lipeilong2012@126.com (P.L.); hucun402@163.com (C.H.)

**Keywords:** ZrCo alloy, element substitution, anti-disproportionation, hydrogen storage performance, storage and delivery system

## Abstract

Hydrogen-induced disproportionation (HID) during the cycles of absorption and desorption leads to a serious decline in the storage capacity of the ZrCo alloy, which has been recognized as the biggest obstacle to its application. Therefore, the prerequisite of a ZrCo application is to solve its anti-disproportionation problem in the field of rapid hydrogen isotope storage. Beyond surface modification and nanoball milling, this work systematically reviews the method of element substitution, which can obviously improve the anti-disproportionation. From a micro angle, as hydrogen atoms that occupy the 8e site in the ZrCoH_3_ lattice are instable and are considered to be the driving force of disproportionation, researchers believe that element substitution by changing the occupation of hydrogen atoms at the 8e site can improve the anti-disproportionation of the alloy. At present, Ti/Nb substitutions for the Zr terminal among substitute elements have an excellent anti-disproportionation performance. In this work, up-to-date research studies on anti-disproportionation and its disproportionation mechanism of the ZrCo alloy are introduced by combining experiments and simulations. Moreover, the optimization of the alloy based on the occupation mechanism of 8e sites is expected to improve the anti-disproportionation of the ZrCo alloy.

## 1. Introduction

Fusion energy has the advantages of huge energy, abundant fuel resources, and low radioactivity, which meets our expectations for future energy. The famous fusion reactor, the International Thermonuclear Experimental Reactor (ITER), was built to research effectiveness, in the sense of technological possibility, using a deuterium–tritium plasma fusion reaction [1,2,3,4,5,6,7,8,9,10,11,12,13]. Due to the scarcity and radioactivity of tritium, and the actual operation of the fusion reactor, deuterium and tritium fuel gas must be quickly supplied to the fuel filling system in a limited time according to the operation scene of the plasma [14,15]. This requires an efficient and safe storage and delivery system (SDS) to ensure the smooth operation of the fusion reactor while avoiding radioactive hazards and waste of resources, and the SDS is a crucial important part of the ITER fuel cycle [16,17,18].

Compared with the general methods of high-pressure gaseous hydrogen storage and low-temperature liquid hydrogen storage, hydrogen storage metallic alloys have shown the ability to provide an excellent combination of economics, efficiency, and safety [19]. Uranium is widely used in the field of hydrogen treatment owing to its favorable characteristics of hydrogen intake and release temperature and pressure, but disadvantages such as radioactivity and spontaneous combustion also limit its application [20,21,22,23,24,25]. The ZrCo alloy has the advantages of good ^3^He trapping, no radioactivity, low absorption pressure at room temperature, moderate hydrogen desorption temperature, and a higher hydrogen absorption and desorption rate [26,27,28,29,30,31,32,33]. In particular, the ZrCo alloy can be held at a moderate temperature to maintain the equilibrium pressure of hydrogen of 100 kPa. Furthermore, it can avoid the damage of thermal radiation and effectively reduce the tritium permeation effect caused by high temperature [2,16,34,35]. As a result, the ZrCo alloy has been selected as one of the important alternative materials for hydrogen storage by ITER.

However, the hydrogen-induced disproportionation (HID) property seriously weakens its hydrogen storage property in the hydrogen absorption and desorption cycle, and it is still a great challenge that cannot be properly solved by far [36,37]. Up-to-date related research studies focusing on the anti-disproportionation of the ZrCo alloy by element substitution were reviewed in this study to tune its mechanism and improve the performance.

## 2. Hydrogen Storage Characteristics of ZrCo

Konishi and Nagasaki et al. [31,38,39] studied the ZrCo alloy and reported it as a hydrogen isotope/hydrogen storage alloy that can replace uranium. Then Shmayda et al. [2] further found that the thermodynamic hydrogen storage properties of the ZrCo alloy and uranium are very similar, but the ZrCo alloy does not have uranium’s radioactivity and its price is reasonable. It was found that, compared to uranium, the ZrCo alloy has a lower hydrogen absorption plateau pressure at room temperature and an appropriate temperature under a higher desorption hydrogen plateau pressure. These two characteristics are of significance for the storage and delivery of hydrogen isotopes, because a high temperature during the process of hydrogen desorption and a high platform pressure during the process of hydrogen absorption increase the possibility of tritium permeation [40,41,42].

In addition, the ZrCo alloy exhibits a good isotope effect, which is beneficial for the separation of the hydrogen isotopes, and the isotope effect is greatly affected by the temperature. The kinetic property test indicates that the absorption rate for deuterium and tritium is slower than that of hydrogen. Moreover, the disproportionation reaction is faster with hydrogen than deuterium, which is well explained by a nucleation and nuclei growth model. However, the maximum extent of disproportionation remains unchanged with different isotopes [10,43,44].

**Figure 1:**
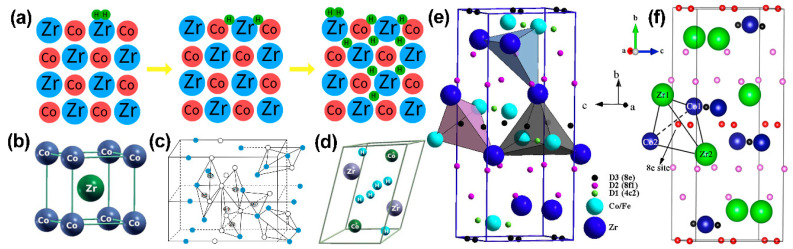
(**a**) Dissociation, adsorption, and permeation of hydrogen molecules; (**b**) crystal structure of ZrCo [46]; (**c**) the 6 types of tetrahedral sites and 2 types of octahedral sites of ZrCoH_3_ are illustrated [10]; (**d**) crystal structure of ZrCoH_3_ [46]; (**e**) crystal structure of ZrCo_0.9_Fe_0.1_D_3_ [47]; (**f**) structure of ZrCoH_3_ [48].

Generally, five steps are required for the ZrCo alloy to absorb hydrogen molecules. The hydrogen adsorption process is as follows: (1) gaseous hydrogen molecules are physically adsorbed onto the metal surface, (2) gaseous hydrogen molecules dissociate into hydrogen atoms and hydrogen atoms are chemically absorbed onto the metal surface, (3) hydrogen atoms permeate into the metal lattice, (4) hydrogen atoms diffuse into the metal interior through the hydride layer, and (5) hydrogen atoms further form a hydride at the metal–hydride interface [45]. As shown in Equation (1) and Figure 1:

2ZrCo + 3H_2_ ↔ 2ZrCoH_3_ (1)

The space group of ZrCo is Pm-3 m. Under ideal conditions, the maximum content of H in the ZrCo–H_2_ system is that each Zr atom matches 3 H atoms, and the space group of ZrCoH_3_ is Cmcm.

The dehydrogenation process involves the opposite steps mentioned above. In fact, the hydrogen absorption and desorption rate of the ZrCo alloy is lower than that of uranium. Uranium can adsorb 90% of hydrogen in 200 s, whereas the ZrCo alloy takes 620 s for the same process, and this is one of the reasons why it cannot be applied to the ITER [2]. Another reason is that in steps (4) and (5), when the temperature is higher than 573 K, the disproportionation during de-/hydrogenation reactions between ZrCo and H_2_ occurs as represented by Equations (2) and (3) [49,50,51,52]. The higher the temperature and hydrogen pressure, the greater the rate and degree of disproportionation reaction can become. Hydrogen-induced disproportionation of the ZrCo alloy was initially reported by Shmayda [2]. The study of Konishi et al. showed that the ZrCo alloy disproportionates obviously when the temperature is higher than 400 °C and the hydrogen pressure is higher than the equilibrium hydrogen pressure, and the complete disproportionation only takes 5 h at 500 °C [53]. Hara et al. also showed that with the increase in temperature, disproportionation accelerates when the temperature is lower than 815 K, and starts to slow down when it exceeds 815 K. Furthermore, the disproportionation rate has a relationship with the initial hydrogen pressure of 3/2 power [44], as follows:

2ZrCoH_3_ → ZrH_2_ + ZrCo_2_ + 2H_2_ (2)

2ZrCo + H_2_ → ZrH_2_ + ZrCo_2_ (3)

The disproportionation product ZrCo_2_ loses its hydrogen absorption capacity; meanwhile, the other product, ZrH_2_, is more stable than ZrCoH_3_ and needs a temperature much higher than 500 °C to completely release hydrogen atoms [54]. There is no doubt that the disproportionation reaction breaks the reversible process of the hydrogen absorption and desorption cycle and weakens the hydrogen storage capacity of the alloy. Up-to-date research work has studied the occupation mechanism of the H atom to improve anti-disproportionation. In fact, the crystal structure (orthorhombic system) of ZrCoH_3_ is similar to that of ZrNiH_3_ [55,56,57]. The tetrahedral sites in ZrCoH_3_ lattices can be divided into six types according to the difference of the nearest neighbor coordination atoms of the hydrogen atoms, i.e., 4c_1_, 4c_2_, 8f_1_, 8g_1_, 8f_2_, and 8e. Because the sizes of 8f_2_ and 8e sites are too small, they are called unstable occupation sites of hydrogen atoms. For example, the distances of 8f_2_–8f_2_ sites, 8f_2_–8e sites, and adjacent 8e–8e sites are 1.1 Å, 1.3 Å, and 1.8 Å, respectively. Thus, hydrogen atoms can only occupy part of them [16]. Ideally, H atoms only occupy sites of 4c_2_ and 8f_1_ in the crystal structure, but this is difficult to achieve in practice. Jat et al. [58] investigated deuterium atoms’ space occupation in the ZrCoD_3_ lattice through neutron diffraction, and confirmed that deuterium atoms did occupy mainly 8f_1_, 4c_2_, and 8e sites, with proportions of 66.9%, 29.3%, and 3.8%, respectively. The structure of ZrCoH_3_ used in calculations is shown in Figure 1f [48,58]. Many studies have shown that the H atoms entering the 8e site in the HID process are the driving force for the disproportionation of the ZrCo alloy [10,48,58,59,60].

In order to solve the disproportionation problem of the ZrCo alloy and improve its dynamic properties, a large number of attempts have been carried out in recent years. The main aspects are as follows: (1) the study of element substitution of the ZrCo alloy and (2) the study of the disproportionation mechanism. In this work, the research progress on ZrCo-based alloys in recent years is reviewed, with an emphasis on the effect of element substitution on anti-disproportionation properties and its deep-seated mechanism.

## 3. Effects of Element Substitution on Anti-Disproportionation

The ZrCo alloy is an excellent hydrogen storage material with low equilibrium pressure at room temperature, high hydrogen absorption content (1.96 wt%), strong safety, and better hydrogen fixation capacity than metal uranium. Its molecular weight is 150.15 g/mol, the melting point is ~1380 °C and the crystal structure is CsCl-type cubic (bcc). The lattice constant and the cell volume are 3.196 Å and 32.65 Å^3^, respectively [42]. It was found that the disproportionation of the ZrCo alloy occurs obviously when the hydrogen desorption temperature is above 350 °C. Generally, with a high hydrogen pressure, the disproportionation rate increases with an increase in temperature. In actual experiments, the desorption temperature required for ZrCo hydride to release hydrogen into a 100 KPa hydrogen pressure atmosphere is 400 °C [2,16]. There is no doubt that, under the condition of satisfying our higher hydrogen pressure transfer rate in SDS, this temperature will induce an obvious disproportionation effect. Although the disproportionation of ZrCo can be regenerated under an ultra-high temperature and vacuum condition, it is not practical in application.

Therefore, in order to restrain the occurrence of disproportionation and explore its mechanism, researchers tried the method of element substitution, with the precondition that the hydrogen absorption and desorption ability of the alloy cannot be destroyed. In fact, researchers have tried many substitute elements (Cu, Cr, Mn, Al, Fe, Ni, Ti, Hf, Sc) [61,62,63,64,65,66,67]. For example, Hf and Zr are in the same main family, and HfCo and ZrCo have the same structure of CsCl. Hf-doped ZrCoH_3_ has a much better ability of anti-disproportionation than ZrCo. However, a Zr_0.8_Ti_0.2_Co bed had a better cyclic hydrogen delivery property than that of both a ZrCo bed and a Zr_0.8_Hf_0.2_Co bed [18,62]. From the perspective of anti-disproportionation, this paper selected four representative substitute elements. ZrCo is an AB-type alloy, and Ti and Nb are A-terminal substitute elements, while Ni and Fe are B-terminal substitute elements. These substitute elements have similar electronic structures to Zr or Co, and it is beneficial to form a single solid-solution phase, thus effectively avoiding the emergence of a multi-hydrogen pressure platform. At present, the method of element substitution for the ZrCo alloy is generally arc melting or magnetic levitation melting in vacuum, but when arc melting is used to replace Co with Ni or Fe, a small amount of the ZrCo_2_ phase is produced in addition to the main phase [16,47,68].

### 3.1. Replacing Zr with Nb and Ti

**Figure 2:**
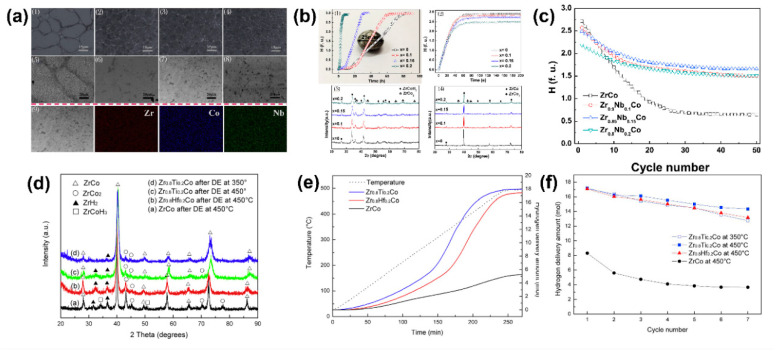
(**a**) Metallographic (1–4) and Back-scattered electron imaging (BSE) (5–8) micrographs of Zr_1–x_Nb_x_Co alloys: (1), (5) x = 0; (2), (6) x = 0.1; (3), (7) x = 0.15; (4), (8) x = 0.2. Energy dispersive X-ray spectrometer (EDS) mapping micrographs (9) of the Zr_0.85_Nb_0.15_Co alloy; (**b**) ingot morphology and initial activation curves of Zr_1__–__x_Nb_x_Co samples (1) and hydriding kinetics curves of activated Zr_1__–__x_Nb_x_Co samples (2). X-ray diffraction (XRD) patterns of Zr_1__–__x_Nb_x_Co–H hydride samples after activation (3) and after dehydrogenation (4); (**c**) comparison of the cyclic hydrogen desorption capacity of Zr_1–x_Nb_x_Co–H (x = 0–0.2) at 350 °C [69]; (**d**) XRD patterns of the samples loaded in the various beds after hydrogen delivery for the seventh cycle; (**e**) temperature-programmed hydrogen delivery curves of the ZrCo bed, Zr_0.8_Hf_0.2_Co bed, and Zr_0.8_Ti_0.2_Co bed from room temperature to 500 °C at a 2 °C/min heating rate; (**f**) comparison of hydrogen delivery amounts of the ZrCo bed, Zr_0.8_Ti_0.2_Co bed, and Zr_0.8_Hf_0.2_Co bed for seven cycles at 350 °C and 450 °C [18].

Nb was used to substitute Zr in ZrCoH_3_ by the magnetic induction suspension melting method; the effects of Nb substitution on the microstructure and initial activation dehydrogenation thermodynamics are shown in Figure 2a,b. From the perspective of anti-disproportionation, the optimal solution is obtained when the amount of Nb in the Zr_1–x_Nb_x_Co (x = 0.1, 0.15, 0.2) is X = 0.20. The atomic radius of Nb is 1.43 Å, which is smaller than that of Zr (1.62 Å). It can be seen from Table 1 that the lattice parameters and cell volume of the ZrCo phase decrease as the Nb content increases. The initial activation period decreases from 87.79 h for ZrCo to 8.08 h for Zr_0.8_Nb_0.2_Co. The pressure of the desorption platform increases significantly from 0.197 bar (x = 0) to 0.85 bar (x = 0.2) at 350 °C. The experimental results, after 50 hydrogen absorption and desorption cycles at 350 °C, are shown in Figure 2c. The hydrogen storage capacity retention rate of the Zr_0.8_Nb_0.2_Co alloy is 68.5%, and it is much higher than that of the ZrCo alloy. From the point of view of thermodynamics, it can also be seen that the disproportionation energy of the new alloy increases from 147.8 kJ/mol for ZrCo to 182.1 kJ/mol for Zr_0.85_Nb_0.15_Co [69].

Ti is another substitute element for Zr to efficiently improve the hydrogen storage properties of the ZrCo alloy; the effects of Ti substitution on the microstructure and temperature-programmed hydrogen delivery curves are shown in Figure 2d,e. When the Ti content of Zr_1–x_Ti_x_Co (x = 0.1, 0.2, 0.3) is x = 0.2, the hydrogen desorption platform pressure can reach 1 bar at 350 degrees, and the temperature is much lower than the equilibrium temperature of the ZrCo alloy at a platform pressure of 1 bar [16,18,70,71]. As shown in Figure 2f, after seven hydrogen absorption and desorption cycles at 450 °C, the hydrogen delivery amount of Zr_0.8_Ti_0.2_Co decreases from 17.18 mol to 14.34 mol. In the study of its principle, it was found that Ti substitution reduces the hydrogen occupation of the 8e site and increases the corresponding Zr–H distance [48]. The lattice parameters and cell volume also decrease as the Ti content increases, as shown in Table 1 [9,72,73,74].

### 3.2. Replacing Co with Ni and Fe

**Figure 3:**
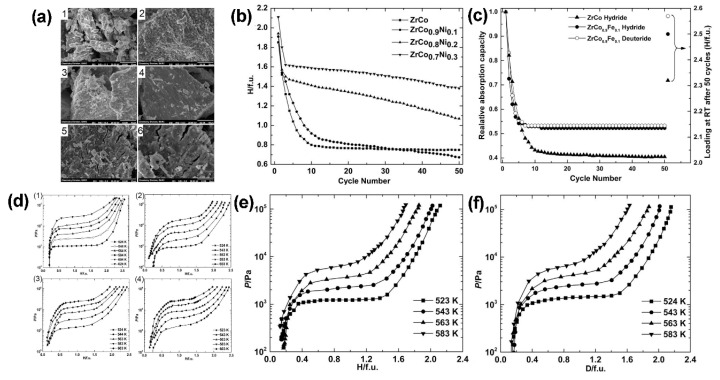
(**a**) Scanning electron microscope (SEM) images of ZrCo_1–x_Ni_x_ alloys at magnifications of 5000×: (1) x = 0, (2) x = 0.1, (3) x = 0.2, (4) x = 0.3, (5) and (6) hydride of ZrCo at magnifications of 5000× and 10,000×, respectively; (**b**) effect of hydrogen absorption–desorption cycles on the storage capacity of ZrCo_1–x_Ni_x_ alloys at 583 K [68]; (**c**) effect of hydrogen/deuterium absorption–desorption cycles on the storage capacity of ZrCo_0.9_Fe_0.1_ and ZrCo alloys at 583 K [47]; (**d**) hydrogen desorption pressure–composition isotherms for ZrCo_1–x_Ni_x_–H_2_ systems: (1) x = 0, (2) x = 0.1, (3) x = 0.2, and (4) x = 0.3 [68]; (**e**) hydrogen desorption pressure–composition isotherms for ZrCo_0.9_Fe_0.1_–H_2_ systems; (**f**) deuterium desorption pressure–composition isotherms for ZrCo_0.9_Fe_0.1_–D_2_ systems [47].

Ni was used to replace Co in ZrCoH_3_; the effects of Ni substitution for Co on the microstructure and hydrogen desorption pressure–composition isotherms are shown in Figure 3a,d. In the Ni-doped alloy ZrCo_1–x_Ni_x_ (x = 0.1, 0.2, 0.3), x = 0.3 is the optimal solution. When the temperature is 583 K, after 50 hydrogen absorption–desorption cycles, the ultimate hydrogen storage capacity is shown in Figure 3b: ZrCo_0.7_Ni_0.3_ > ZrCo_0.8_Ni_0.2_ > ZrCo > ZrCo_0.9_Ni_0.1_. The XRD test shows that the lattice parameters and cell volume increase as the Ni content increased, as presented in Table 1 [69,76]. As the Ni content increases, the hydrogen atom occupancy of the 8e site decreases from ~3.8% for ZrCoD_3_ to ~2.5% for ZrCo_0.7_Ni_0.3_D_3_, and the Zr–D distance of ZrCoD_3_ increases from 1.937 Å to 2.022 Å (ZrCo_0.7_Ni_0.3_D_3_) [58].

Fe is another substitute element for Co; the effects of Fe substitution for Co on the pressure–composition isotherms and anti-disproportionation are shown in Figure 3c,e,f. After 50 hydrogen/deuterium absorption–desorption cycles at 583 K, the storage capacity of the ZrCo–H_2_ system decreases by 19.2%, while the storage capacities of the ZrCo_0.9_Fe_0.1_–H_2_ system and the ZrCo_0.9_Fe_0.1_–D_2_ system are 8.5% and 10.1%, respectively. In general, the final storage capacity of the ZrCo_0.9_Fe_0.1_ alloy is significantly higher than that of the ZrCo alloy, which is similar to that in Ni–doped ZrCoH_3_. The lattice parameters and cell volume of the ZrCo_0.9_Fe_0.1_ alloy are larger than those of the ZrCo alloy; however, as shown in Table 2, the occupancy of the 8e site significantly reduces from 3.8% to 1.8%, and the length of Zr–D (8e) also increases from 1.937 Å to 2.201 Å. Both of them significantly enhance the hydrogen anti-disproportionation of the alloy [47].

### 3.3. Double Doping of Ti/Fe and Ti/Ni

**Figure 4:**
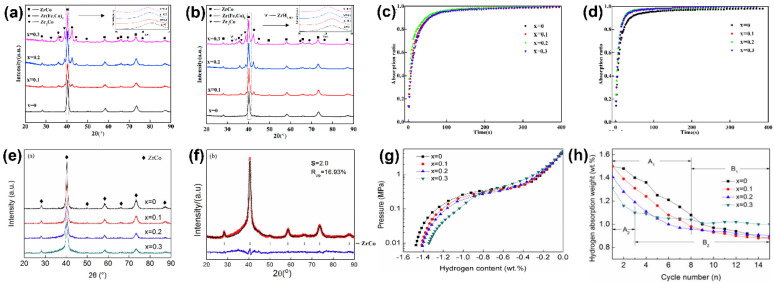
(**a**) XRD patterns of Zr_0.8_Ti_0.2_Co_1–x_Fe_x_ (x = 0–0.3) alloys after arc melting; (**b**) XRD patterns of Zr_0.8_Ti_0.2_Co_1–x_Fe_x_ (x = 0–0.3) alloys after 5 hydrogen absorption–desorption cycles; (**c**) the second hydriding kinetic curve of Zr_0.8_Ti_0.2_Co_1–x_Fe_x_ (x = 0–0.3) alloys at 573 K; (**d**) the fifth hydriding kinetic curve of Zr_0.8_Ti_0.2_Co_1–x_Fe_x_ (x = 0–0.3) alloys at 573 K [75]; (**e**) XRD patterns of Zr_0.8_Ti_0.2_Co_1–x_Ni_x_ (x = 0–0.3) alloys; (**f**) XRD pattern and Rietveld analysis pattern of Zr_0.8_Ti_0.2_Co_0.7_Ni_0.3_; (**g**) desorption Pressure-composition-temperature (PCT) curves for Zr_0.8_Ti_0.2_Co_1–x_Ni_x_–H_2_ (x = 0–0.3) systems at 673 K; (**h**) cyclic stability curve of the Zr_0.8_Ti_0.2_Co_1–x_Ni_x_ alloy at 573 K [77].

Researchers designed an alloy with a composition of Zr_0.8_Ti_0.2_Co_1–x_Fe_x_ (x = 0.1–0.3) to further improve the overall hydrogen storage properties of ZrCo-based alloys. The microstructure and cyclic hydriding kinetic property are shown in Figure 4a–d. It was found that after five absorption–desorption cycles at 573 K, the hydrogen storage retention rate in Fe-doped ZrCoH_3_ is 87% (x = 0.1), 82% (x = 0.2), and 80% (x = 0.3), whereas the highest retention rate in undoped ZrCoH_3_ is 88%. The atomic radius of the Fe element is 1.27 Å, which is slightly larger than that of the Co element. As shown in Table 1, we can see that the unit cell volume and lattice parameters of the ZrCo phase of the Zr_0.8_Ti_0.2_Co_1–x_Fe_x_ alloy increase gradually as the Fe content increases. In fact, it is extremely difficult to prepare a single phase of ZrCo, which is accompanied by the formation of the ZrCo_2_ phase. XRD patterns show that cubic phase ZrCo and secondary Laves phase of Zr(Co,Fe)_2_ and Zr_2_Co are formed in the Zr_0.8_Ti_0.2_Co_1–x_Fe_x_ alloy. Moreover, the contents of the secondary phase increase with the increase in Fe substitution. Although the Laves phase provides abundant phase interfaces as hydrogen diffusion channels to improve the hydriding kinetic property, the anti-disproportionation and hydrogen storage capacity are weakened at the same time [75].

Similarly, the substitution of the single elements Ni for Co and Ti for Zr can improve the cyclic stability of the ZrCo alloy to some extent. Therefore, Zr_0.8_Ti_0.2_Co_1–x_Ni_x_ (x = 0–0.3) alloys were prepared by vacuum arc melting, as shown in Figure 4e–h. After 15 hydrogen absorption–desorption cycles at 573 K, the hydrogen storage retention rates are 58.7% (x = 0), 58.7% (x = 0.1), 63.8% (x = 0.2), and 76.3% (x = 0.3) [77].

It can be seen that the Ni substitution of Zr_0.8_Ti_0.2_Co further improves its anti-disproportionation performance. However, the hydrogen desorption curve at 673 K shows that the anti-disproportionation of the alloy is slightly deteriorated by the partial substitution of the Ni element for the Co element. As shown in Table 1, as the Ni content increases, the lattice parameters and cell volume decrease. At 573 K, the curves are clearly characterized by two stages (part A with a rapid decrease and part B with a slow decline in hydrogen absorption capacity). As shown in Figure 4h, the cyclic stability of the alloy is obviously improved with the Ni partial substitution, and Jing et al. believed this was related to the improvement of the anti-pulverization of the alloy. In contrast, the decrease in cyclic stability of the alloy at 673 K indicates that hydrogen disproportionation is the major factor leading to the deterioration of the hydrogen storage properties [77].

### 3.4. Anti-Disproportionation Mechanism of Doping

It is helpful to solve the problem of disproportionation fundamentally, by probing into the internal disproportionation mechanism of ZrCo-based alloys [78]. These first-principle calculations were performed with the density functional theory (DFT) as implemented in the Vienna Ab-initio Simulation Package (VASP). Through summarizing first-principle simulations [46,48,79,80,81,82,83,84,85,86,87,88,89,90,91,92,93,94,95], it has been shown that, in the ZrCo-H_2_ system, the binding of H with Zr (or its substitute elements) shows a strong ion and weak covalence bond, and the binding of H with Co (or its substitute elements) shows a weak ion and strong covalence bond. In addition, the Zr-D distance in the 8e sites of ZrCoD_3_ is smaller than the Zr-D distance of ZrD_2_ [58]. Therefore, when disproportionation occurs, it is more likely to form ZrH_2_. The calculated binding energies of the H atoms at the 4c_2_ and 8f_1_ sites are −0.58 eV and −0.48 eV, respectively. The H binding energy at the 8e site is 0.05 eV (E_1_) or 0.28 eV (E_2_). E_1_ represents the binding energy in which the nearest H (4c_2_) atom of this 8e site diffuses into the 8e site, and E_2_ represents the binding energy in which an additional H atom is inserted into one 8e site. It is evident that the 4c_2_ and 8f_1_ sites are more stable for the H atom than the 8e site. Hence, H atoms occupy only 4c_2_ and 8f_1_ sites under ideal conditions [60]. The number of H atoms occupying 8e is much lower than that of H atoms occupying the other two sites, accounting for only about 4% of the total hydrogen.

**Figure 5:**
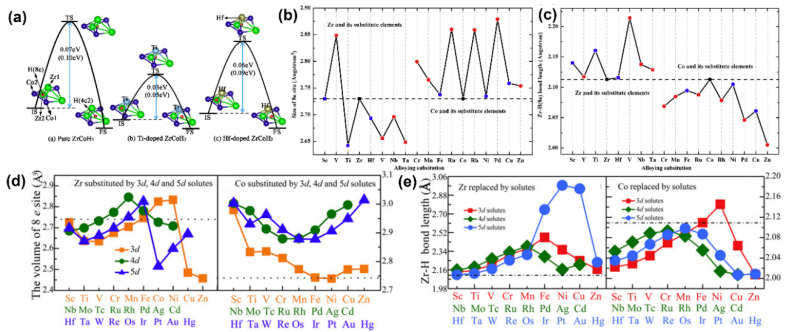
(**a**) Schematic diagram of H diffusion from the 8e site to the 4c_2_ site in undoped, Ti-doped, and Hf-doped ZrCoH_3_; (**b**) sizes of the 8e site in the undoped and doped ZrCoH_3_ crystal; (**c**) the Zr–H (8e) bond length in the tetrahedron of undoped and doped ZrCoH_3_ with the 8e site occupied by a H atom [60]; (**d**) volumes of the tetrahedron when one of the Zr/Co atoms in the tetrahedron is substituted by 3d, 4d, and 5d transition alloying solutes; (**e**) the bond lengths of Zr and H at the 8e site (at the center of the tetrahedron) when one of the Zr/Co atoms in the tetrahedron is substituted by 3d, 4d, and 5d transition alloying solutes [95].

The relationship between the size of the 8e site and the substitution atom can be seen from the simulation in Figure 5b,d. The substitution of the Zr element is favorable for improving the anti-disproportionation of the ZrCo alloy, whereas the substitution from Co may not be suitable to enhance the anti-disproportionation [60,95]. Obviously, if the volume of the 8e site is small, it is hard for the H atom to diffuse into the 8e site, resulting in a significant decrease in the proportion of hydrogen atoms in the 8e site. The H diffusion process in undoped, and doped ZrCoH_3_ is investigated in Figure 5a. Nonetheless, from the above introduction of the Ni and Fe substitution experiments, we can conclude that the Co element substitution also enhances the ability of anti-disproportionation. Therefore, there must be other factors contributing to the anti-disproportionation.

The Zr–H (8e) bond length of ZrCoH_3_ plays a key role in the anti-disproportionation process; the longer the distance of Zr–H (8e), the stronger the inhibition of hydrogen disproportionation can become [47,48,58,60,96]. This can also be seen from the simulation results shown in Figure 5c,e. The length of the Zr–H (8e) bond in the Zr-substituted ZrCoH_3_ is longer than that in the undoped ZrCoH_3_, whereas the length of the Zr–H (8e) bond in the Co-substituted ZrCoH_3_ is shorter than that in the undoped ZrCoH_3_ [60].

At the same time, the occupancy of H (8e) is also an important factor in improving the ability of anti-disproportionation [91]. For instance, Fe substitution has the larger size of 8e and the shorter length of the Zr–H (8e) from the simulation results (Figure 5b,c). Theoretically, Fe-substituted ZrCoH_3_ should decrease the anti-disproportionation performance. Nonetheless, Jat et al. [47] found that the ZrCo_0.9_Fe_0.1_ alloy has better anti-disproportionation than the ZrCo alloy. The neutron diffraction studies reveal that the occupancy of H (8e) of ZrCo_0.9_Fe_0.1_D_3_ is 1.8%, which is lower than ~3.8% of ZrCoD_3_. The lower occupancy of H (8e) explains the higher durability of the ZrCo_0.9_Fe_0.1_ alloy against disproportionation.

The effects of Zr–H (8e) spacing, 8e site size, and occupancy of H (8e) on the anti-disproportionation performance are likewise relatively consistent with the above experimental results.

## 4. Conclusions and Outlook

This review presents an overview of the anti-disproportionation of ZrCo-based alloys along with a discussion of the disproportionation mechanism. At present, the performance of the ZrCo-based alloy cannot meet requirements, and it is unable to meet the high-pressure transportation required by the storage and delivery system at the current working temperature that suppresses disproportionation, or eliminate the disproportionation at the high temperature that meets the storage and transport rate. Obviously, element substitution is the most tried and promising way to improve the anti-disproportionation of the ZrCo alloy, compared to other methods [64,67,97,98,99,100,101,102,103,104]. To date, many experiments have shown that samples with smaller lattice parameters and cell volume have the expected anti-disproportionation ability. Nevertheless, it is only the appearance that describes the anti-disproportionation performance, and it is not the determinant of a stronger anti-disproportionation performance caused by element substitution [105,106,107]. For instance, when the Ni element substitutes for the Co element, the doped sample has a better cyclic stability and anti-disproportionation performance, but the lattice parameters and cell volume increase with Ni substitution. Obviously, this does not correspond to the above expectation, the fundamental reasons of the anti-disproportionation being that the occupancy of H (8e) decreases from ~3.8% to ~2.5% (ZrCo_0.7_Ni_0.3_D_3_), and the Zr–D (8e) distance increases from 1.937 Å to 2.022 Å (ZrCo_0.7_Ni_0.3_D_3_) compared with ZrCoD_3_ [58]. In other words, the length of the Zr–H (8e) bond is an important factor affecting the disproportionation activation energy; similarly, the size of the 8e site reflects the ability of the H atom to diffuse out and diffuse into the 8e site, and the occupancy of H (8e) is also an important factor in improving the ability of anti-disproportionation. Hence, the instability occupied by the hydrogen atom on the 8e site can be seen as the driving force of disproportionation.

However, where does the H atom of the 8e site come from? The answer is perhaps the 4c_2_ site or the 8f_1_ site, shown in Figure 1c or Figure 1e,f. It was found from Table 2 that Ni/Fe substitutions decrease the occupancy of H (8e), while the occupancy of H (4c_2_) and H (8f_1_) increases. We can attempt to influence the occupancy of H (8e) by changing adjacent sites, such as the distance of H (8e) and H (4c_2_). It can be predicted that, through an in-depth study of the disproportionation mechanism, the optimal element substitution can be selected by calculation and experimental methods to suppress the hydrogen atoms occupying the 8e sites, so as to further improve the anti-disproportionation effect of ZrCo-based alloys.

## Figures and Tables

**Table 1:** Lattice parameters and cell volumes of ZrCo-based alloys measured by XRD.

Alloy	Main Constituent Phase Lattice Parameters (ZrCo phase) (Å)	Cell Volume (Å3)	Reference
ZrCo	3.1957 ± 0.0001	32.637 ± 0.002	[68]
ZrCo_0.9_Ni_0.1_	3.1971 ± 0.0042	32.678 ± 0.074	
ZrCo_0.8_Ni_0.2_	3.1982 ± 0.0041	32.713 ± 0.072	
ZrCo_0.7_Ni_0.3_	3.1988 ± 0.0020	32.731 ± 0.0035	
Zr_0.9_Ti_0.1_Co	3.1788 ± 0.0001	32.120 ± 0.002	[9]
Zr_0.8_Ti_0.2_Co	3.1591 ± 0.0001	31.528 ± 0.002	
Zr_0.7_Ti_0.3_Co	3.1404 ± 0.0001	30.972 ± 0.002	
Zr_0.9_Nb_0.1_Co	3.1898	32.49	[69]
Zr_0.85_Nb_0.15_Co	3.1836	32.34	
Zr_0.8_Nb_0.2_Co	3.1773	32.2	
Zr_0.8_Ti_0.2_Co_0.9_Ni_0.1_	3.163	31.645	[75]
Zr_0.8_Ti_0.2_Co_0.8_Ni_0.2_	3.161	31.581	
Zr_0.8_Ti_0.2_Co_0.7_Ni_0.3_	3.159	31.524	
Zr_0.8_Ti_0.2_Co_0.9_Fe_0.1_	3.1652	31.7105	
Zr_0.8_Ti_0.2_Co_0.8_Fe_0.2_	3.1655	31.7195	
Zr_0.8_Ti_0.2_Co_0.7_Fe_0.3_	3.1707	31.8761	
ZrCo_0.9_Fe_0.1_	3.1959	32.642	[47]

**Table 2:** Rietveld optimization results for neutron diffraction data of ZrCo_1–x_Fe_x_ and ZrCo_1–x_Ni_x_ hydride [47,58].

Parameters		x in ZrCo_1-x_Fe_x_ Deuterides		x in ZrCo_1–x_Ni_x_ Deuterides		
		0	0.1	0.1	0.2	0.3
D1 (4c_2_) (0 y 1/4)	y	0.9269 ± 0.0039	0.9279 ± 0.0003	0.9300 ± 0.0005	0.9282 ± 0.0003	0.9291 ± 0.0003
Occupancy (%)		29.3	29.9	30.8	30.1	31.1
D2 (8f_1_) (0 y z)	y	0.3119 ± 0.002	0.3119 ± 0.0002	0.3125 ± 0.0003	0.3122 ± 0.0002	0.3119 ± 0.0002
	z	0.5047 ± 0.0006	0.5041 ± 0.0005	0.5047 ± 0.0007	0.5062 ± 0.0004	0.5053 ± 0.0004
Occupancy (%)		66.9	68.3	66.4	67.2	66.4
D3 (8e) (x 0 0)	x	0.2219 ± 0.0081	0.3725 ± 0.0139	0.2243 ± 0.0139	0.2549 ± 0.0088	0.2637 ± 0.0095
Occupancy (%)		3.8	1.8	2.8	2.7	2.5
Zr-D distance (Å)		1.937 ± 0.012	2.201 ± 0.030	1.940 ± 0.020	1.994 ± 0.014	2.022 ± 0.015
R_p_ (%)		3.29	1.93	6.7	1.81	2.13
R_wp_ (%)		4.15	2.45	9.86	2.36	2.89
R_exp_ (%)		3.61	1.81	2.46	1.32	1.4
